# HBV Drug Resistance Substitutions Existed before the Clinical Approval of Nucleos(t)ide Analogues: A Bioinformatic Analysis by GenBank Data Mining

**DOI:** 10.3390/v9080199

**Published:** 2017-07-27

**Authors:** Xizhan Xu, Kuanhui Xiang, Mingze Su, Yao Li, Wei Ji, Yutang Li, Hui Zhuang, Tong Li

**Affiliations:** Department of Microbiology and Center of Infectious Disease, School of Basic Medical Sciences, Peking University Health Science Center, Beijing 100191, China; xuxizhan2017@163.com (X.X.); xiangkuanhui174@126.com (K.X.); sumingze88@163.com (M.S.); yao.lee@foxmail.com (Y.L.); jiwei_yunlong@126.com (W.J.); liyutang@bjmu.edu.cn (Y.L.)

**Keywords:** hepatitis B virus, reverse transcriptase, nucleos(t)ides analogue resistance, substitution, pre-existing, naturally occurring

## Abstract

Naturally occurring nucleos(t)ide analogue resistance (NUCr) substitution frequencies in the reverse transcriptase (RT) of the hepatitis B virus (HBV) were studied extensively after the clinical approval of nucleos(t)ide analogues (NUCs; year of approval 1998). We aimed to study NUCr substitutions in HBV RT sequences obtained before 1998 and better understand the evolution of RT sequences without NUC pressures. Our strategy was to retrieve HBV sequences from GenBank deposited before 1998. The initial search used the keywords “hepatitis B virus” or “HBV” and 1139 sequences were found. Data analyses included information extraction: sequence quality control and amino acid substitution analysis on 8 primary NUCr and 3 secondary substitution codons. Three hundred and ninety-four RT-containing sequences of 8 genotypes from 25 countries in 4 continents were selected. Twenty-seven (6.9%) sequences were found to harbor substitutions at NUCr-related codons. Secondary substitutions (rtL80V and rtV173G/A/L) occurred more frequently than primary NUCr substitutions (rtI169L; rtA181G; T184A/S; rtS202T/R; rtM204L and rtM250K). Typical amino acid substitutions associated with NUCr were of rtL80V, rtV173L and rtT184A/S. We confirm the presence of naturally occurring typical HBV NUCr substitutions with very low frequencies, and secondary substitutions are more likely to occur than primary NUCr substitutions without the selective pressure of NUCs.

## 1. Introduction

Hepatitis B virus (HBV) infection remains a serious public health issue [1]. Six antiviral nucleos(t)ide analogues (NUCs), namely lamivudine (LAM), adefovir dipivoxil (ADV), entecavir (ETV), telbivudine (LdT), tenofovir disoproxil fumarate (TDF) and tenofovir alafenamide fumarate (TAF) have been successively approved for treating chronic hepatitis B (CHB) since 1998 [2,3,4,5]. They are all HBV polymerase/reverse transcriptase (RT) inhibitors and can efficiently suppress HBV replication. However, because RT lacks proofreading activity, HBV replication is error-prone and leads to the incorporation of many mutations in its genome [6]. The clinical use of NUCs was limited by the development of NUC resistance (NUCr) substitutions in RT sequences during long-term therapy until the emergence of ETV, TDF and TAF, which have high genetic barriers to resistance [2,7]. However, pre-existing NUCr substitutions can be detected in treatment-naïve patients, which have been shown to affect the antiviral responses [8,9,10].

According to several clinical practice guidelines and authoritative reviews, NUCr substitutions can be classified into two categories: primary NUCr substitutions at 8 codons (rtI169T, rtA181T/V, rtT184A/C/F/G/I/L/M/S, rtA194T, rtS202C/G/I, rtM204I/V, rtN236T and rtM250I/L/V) and secondary substitutions at 3 codons in RT (rtL80I/V, rtV173L and rtL180M), which are often found in treated patients and termed as classic amino acid (AA) substitutions [2,4,11,12,13,14,15]. Primary NUCr substitutions are known to reduce HBV susceptibility to NUCs but with the cost of impaired viral replication, which have been well characterized in vitro [11]. Secondary substitutions contribute to restoring the functional defects of RT caused by primary NUCr substitutions [14,16]. The relationship between AA substitutions at these codons and NUCr was extensively studied after 1998 [2,4]. Resistance to LAM or LdT is conferred by rtM204I/V, which is often accompanied by rtL80I/V, rtL180M and/or rtV173L. Resistance to ADV is linked to rtA181T/V or rtN236T. Substitution of rtA181T/V can also cause decreased susceptibility to LAM or LdT. Resistance to ETV needs a combination of substitutions of rtM204I/V and rtL180M plus one of the following substitutions: rtI169T, rtT184A/C/F/G/I/L/M/S, rtS202C/G/I or rtM250I/L/V. Resistance to TDF/TAF has not been well elucidated yet [2,4,11]. LAM, the first approved oral NUC in 1998, has been excluded by several clinical practice guidelines as a first-line antiviral agent due to the high frequency of NUCr substitution. ETV and TDF/TAF have the lowest rates of NUCr and are recommended as the first-line antiviral NUCs for CHB today [5,12,13].

In addition, through the review of the published literature and a systematic analysis of potential substitutions associated with NUCr, we also summarized some non-classic substitutions in the RT domain of HBV polymerase that appeared sporadically in clinical practice and might contribute to decreasing susceptibility to NUCs or improve the virus fitness (Table 1). For example, Hayashi et al. [17] characterized two novel substitutions, rtI163V and rtA186T, in an ETV-refractory patient. The rtA186T substitution was responsible for ETV resistance and significantly reduced viral replication efficacy, while rtI163V substitution could sustain viral fitness and rescue the variant. Shirvani-Dastgerdi et al. [18] identified rtS78T as being partially responsible for multidrug resistance to ETV and TDF in two patients receiving ETV-TDF combination therapy. However, the emergence of NUCr caused by these non-classic substitutions remains limited in clinical practice.

Previous studies have reported that HBV NUCr substitutions might pre-exist in the RT region in treatment-naïve patients [8,9,10]. They might come from naturally occurring substitutions or horizontal transmission from NUC-exposed patients. However, the pre-existing NUCr substitution frequencies reported varied from 0% to 35.7% in previous studies [8,9,10,11,19,20,21,22,23,24]. For instance, our group has reported that no pre-existing NUCr substitution was observed among 192 treatment-naïve Chinese patients using a population-sequencing approach, while Tan et al. found a relatively high incidence of rtM204I/V substitution (23.3%) in 1042 untreated CHB patients [10,11]. According to a recent meta-analysis of 106 studies involving 12212 treatment-naïve CHB patients, the summarized incidence of NUCr substitution frequency worldwide was 5.73% (95% confidence interval: 4.85–6.61%), which was obviously lower than the abnormally high prevalence of NUCr given by Tan et al. [10,24]. We speculate that such a wide range of NUCr substitution frequencies in treatment-naïve patients might be due to discrepancies of the following factors: study design, sample sizes/sources, substitution testing methods, and so on [20,25]. More importantly, several key factors can largely affect the results, leading to inappropriate interpretation of the true prevalence of pre-existing NUCr substitutions. First, patients enrolled in some studies might receive antiviral therapy without awareness of the exposure, i.e., lack of critical validation for treatment-naïvety at enrollment [20]. Second, treatment-naïve patients might be infected with NUCr HBV strains through horizontal transmission. Third, different studies might focus on investigating inconsistent NUCr substitution codons. For example, initial NUCr studies often investigated rtM204I/V substitution, while more AA substitution codons associated with NUCr in the RT domain have been included in more recent studies [10,26]. Characterizing NUCr substitutions in patients before the clinical approval of NUCs is a good way to illustrate if the pre-existing NUCr substitutions are naturally occurring. However, the serum samples collected before 1998 are difficult to obtain.

In this study, we analyzed HBV RT sequences before the clinical approval of NUCs to characterize the pre-existing NUCr substitutions by using publically available GenBank data. Our study focuses on analyzing the classic NUCr substitutions recommended by several clinical practice guidelines. These results can provide critical information regarding pre-existing (largely due to naturally occurring) NUCr substitutions in HBV RT sequences and a better understanding of HBV evolution without selective pressure of NUCs.

## 2. Materials and Methods

### 2.1. Sequence Search and Qualification Strategy

We performed a GenBank database (https://www.ncbi.nlm.nih.gov/nuccore) search using keywords “hepatitis B virus” or “HBV” to retrieve sequences deposited before December 1998, the year just before the clinical approval of NUCs. Each sequence with a unique accession number was retained after manually removing the redundant sequences by checking duplicate sequence accession numbers. The following sequences were then excluded: (1) artificial synthetic constructs; (2) chromosomally integrated HBV sequences with human genes; (3) non-human HBV sequences; (4) other types of hepatitis virus sequences (hepatitis A virus, hepatitis C virus, hepatitis D virus or hepatitis E virus); (5) expression vector sequences; (6) sequences from patients co-infected with HBV/human immunodeficiency virus (HIV) receiving antiviral therapy against HIV; (7) sequences from CHB patients who participated in the clinical trials (phase I, II and III) for LAM; and (8) HBV sequences without the RT region. After exclusion, the rest of the sequences containing the HBV RT region were qualified for further analysis.

### 2.2. Data Extraction

The selection of each sequence and data extraction were reviewed and performed independently by two authors (X.X. and K.X.). For each qualified sequence, the following data were extracted: the GenBank accession number, related nucleotide sequence, sequence release year, sample origin, and sequence length. References related to specific sequences were thoroughly analyzed to collect the relevant information if the GenBank records did not provide it.

### 2.3. HBV Genotyping and Detection of Recombinant Sequences

HBV genotypes were identified using the National Center for Biotechnology Information (NCBI) viral genotyping tool (http://www.ncbi.nlm.nih.gov/projects/genotyping/formpage.cgi). The results were reconfirmed using the online software tool HBV STAR (http://www.vgb.ucl.ac.uk/starn.shtml) [52]. An online tool jpHMM (http://jphmm.gobics.de/submission_HBV) designed for viruses with circular genomes was used to predict genotype recombination [53].

### 2.4. Systematic Analysis of Potential NUCr Substitutions

Potential AA substitutions in RT domain of HBV polymerase associated with NUCr were listed in Table 1. This table was modified from Menéndez-Arias et al. [4] and Liu et al. [11], and updated according to the latest literature. All these AA substitutions have been reported as being selected during NUCs therapy and verified by in vitro phenotypic data.

### 2.5. HBV Drug Resistance Substitution Analysis

HBV RT sequences were extracted from the qualified HBV sequences, which contained various lengths of the RT region. Multiple RT nucleotide sequences were aligned using BioEdit7.0 software (Ibis Biosciences, Carlsbad, CA, USA). All RT nucleotide sequences were translated in frame to AA sequences. Each genetic codon harboring degenerate base was manually translated to the corresponding AA. Classic substitutions at 11 codons that have been often suggested for monitoring NUCr in treated patients were analyzed in this study. The obtained AA sequences were compared with the consensus RT AA sequences to identify the NUCr substitutions at rtL80, rtI169, rtV173, rtL180, rtA181, rtT184, rtA194, rtS202, rtM204, rtN236 and rtM250 [2,54,55,56]. The codons that were mixed with wild-type and substituted AAs were recorded as indicating the presence of a substitution, which might reflect the presence of HBV quasispecies [11,54]. All sequences with NUCr substitutions were further determined by an HBV drug resistance interpretation online tool (http://www.hiv-grade.de/HBV_grade/deployed/grade.pl?program=HBValg).

### 2.6. Statistical Analysis

Statistical analysis was performed using SPSS 20.0 software (IBM, Armonk, NY, USA). Chi-square tests were used for categorical data. *p* Values were calculated with the two-tailed method. *p* Value < 0.05 was considered statistically significant.

## 3. Results

### 3.1. Selection for RT-Containing HBV Sequences

**Figure 1:**
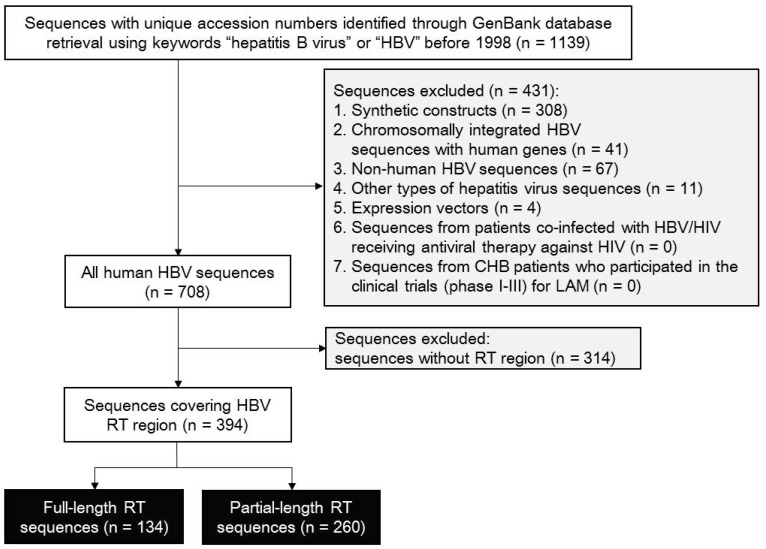
Flowchart showing the process of searching and screening for HBV RT sequences used in this study. The grey boxes show the excluded sequences, while the black boxes show those that qualified for analyses. CHB: chronic hepatitis B; HBV: hepatitis B virus; HIV: human immunodeficiency virus; LAM: lamivudine; RT, reverse transcriptase.

A total of 1139 sequences with unique accession number were found from the GenBank nucleotide database using the keywords “hepatitis B virus” or “HBV” before December 1998. The sequence exclusion and inclusion strategy and processing were illustrated by the flowchart in Figure 1. Seven hundred and eight HBV sequences were identified, in which 314 sequences were excluded because they did not harbor an RT region. Finally, 394 RT-containing (full-length: 134; partial-length: 260) sequences were included for further analysis. Background information (i.e., accession numbers, release time, origin, RT coverage and sequence length) for all qualified sequences is presented in Supplementary Materials (Table S1).

### 3.2. Quality Control for HBV RT-Containing Sequences

**Figure 2:**
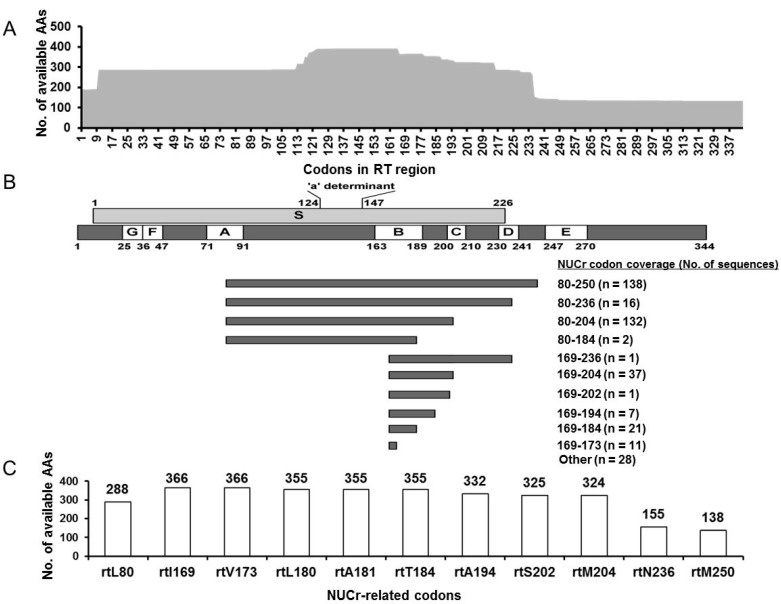
Schematic illustration of the analyzed RT sequences. (**A**) The total number of available AAs at each codon in 394 HBV RT sequences; (**B**) All sequences are clustered based on the different coverage of analyzed NUCr-related codons. For example, “80–250” shows where these sequences include 11 studied NUCr-related codons. “Other” shows where no NUCr-related codons are covered by these sequences; (**C**) The number of available AAs for each of the NUCr-related codons studied is summarized. AA: amino acid; HBV: hepatitis B virus; NUCr: nucleos(t)ide analogue resistance; RT: reverse transcriptase.

The selected RT sequences were further evaluated in terms of their coverage of the studied NUCr-related codons. One hundred and thirty-four sequences were full-length RT sequences, which contained all 11 codons of interest. The other 260 partial-length RT sequences ranged from 23 to 305 AAs in length with a median length of 226 AAs. Figure 2A illustrates the overlapping features of the recruited RT sequences. Our data suggested that most of previous studies appeared to primarily focus on HBV small surface proteins, especially on the ‘a’ determinant region, which is overlapped by the RT region. The RT sequences were further grouped according to their coverage, as shown in Figure 2B. In total, 90.1% (355/394) of the recruited RT sequences carried 5–11 analyzed NUCr substitution codons and only 28 sequences (7.1%) did not cover any studied NUCr codons.

Due to the diversity of the sequences in length and their coverage of the studied codons, the substitution analysis strategy was codon-based rather than sequence-based. The numbers of the available AAs at 11 codons in RT were summarized in Figure 2C. Thus, the substitution frequency was calculated as the percentage of the numbers of substituted AAs with respect to the available AAs at this codon.

### 3.3. RT Sequence Origins and HBV Genotypes

The finally analyzed 394 HBV RT sequences were found from 25 countries within 4 continents. The sequences from Africa, America, Asia and Europe accounted for 4.8%, 23.1%, 41.9% and 30.2%, respectively. Eight HBV genotypes (A: 27.4%; B: 9.4%; C: 31.0%; D: 16.8% E: 2.3%; F: 10.7%; G: 0.5%; H: 0.5%) were found (Figure S1). Moreover, four recombinant sequences were found, which did not affect the NUCr-related codons and could be used for substitution analysis (Figure S2). The geographical distribution of HBV genotypes was consistent with previous reports [57]. Notably, although the dominant genotype in America was F (35.2%), these sequences were mainly from Central and South America (Argentina), not from North America.

### 3.4. NUCr Substitution Analysis

Among the 394 sequences, 27 (6.9%) were found to harbor substitutions at NUCr-related codons (Table 2). Eleven sequences had substitutions at 6 codons associated with primary NUCr, and 16 carried secondary substitutions at 2 codons. However, no combination substitutions at multiple codons were found. No substitution at rtA194, rtN236, or rtL180 was detected.

Moreover, the substitution frequency was significantly higher at the secondary substitution codons (16/1008, 1.6%) than that at the primary NUCr codons (11/2350, 0.5%) (χ^2^ = 11.08, *p* = 0.0009). Among eight codons with detectable substitutions, rtV173 had the highest substitution frequency (9/366, 2.5%), which was significantly higher than the overall substitution frequency (27/3358, 0.8%) (χ^2^ = 9.44, *p* = 0.0021). Interestingly, only rtL80V (*n* = 7), rtV173L (*n* = 2) and rtT184A/S (*n* = 2) were typical NUCr substitutions, whose ability to confer resistance to NUCs have been well elucidated in vitro [2,4,11]. However, the relationship between other atypical AA substitutions with susceptibility to NUCs has not been characterized in vitro, such as rtI169L (not typical rtI169T) and rtM204L (not typical rtM204I/V). Thus, the substitution frequency for typical AA substitutions at NUCr-related codons was as low as 0.3% (11/3358).

### 3.5. Characterization of the RT Sequences Harboring AA Substitutions Associated with NUCr

Table 3 provides the detailed characteristics of 27 RT sequences with AA substitutions at NUCr-related codons. Four sequences (14.8%) belonged to genotype A, 1 (3.7%) belonged to B, 16 (59.3%) belonged to C, and 6 (22.2%) belonged to D. Overall, the NUCr substitutions occurred in 3.7% (4/108) of A, 2.7% (1/37) of B, 13.1% (16/122) of C and 9.1% (6/66) of D genotype sequences. No substitutions at these codons were detected in HBV E to H and recombinant genotypes. The detection frequencies of NUCr substitutions were significantly different among genotypes A to D (*p* = 0.0375). This was mainly due to the significant difference between genotype A (3.7%) and C (13.1%) (*p* = 0.0172). Comparisons between the other two genotypes, i.e., A vs. B, A vs. D, B vs. C, B vs. D and C vs. D, did not show any significant differences (*p* > 0.05).

As shown in Table 3, these 27 sequences were distributed in 12 countries in 4 continents. The substitution frequencies were 5.3% (1/19) for Africa, 2.2% (2/91) for America, 10.3% (17/165) for Asia and 5.9% (7/119) for Europe, respectively (*p* = 0.0930). Asia contributed 63.0% (17/27) of sequences with substitutions at NUCr-related codons, in which 6 and 6 were from China and Japan, respectively (Figure S3).

Moreover, the original studies submitting these 27 sequences were traced and carefully studied. We found that 17 sequences were from published papers, while the others were a direct submission to GenBank. Twelve of these 17 sequences were obtained by clone sequencing. These studies aimed to analyze the sequence variations, mainly focusing on HBV surface proteins (M23808 [59], X14193 [62], M27765 [61], S56208 [64], AF013631 [67], AF043564 [71] and AJ003026-AJ003028 [70]). However, none of them carried out in vitro study to verify whether the identified variants were viable or not.

## 4. Discussion

To the best of our knowledge, this is the first study to analyze HBV NUCr-related substitutions from HBV RT sequences obtained before the clinical approval of NUCs. Our results demonstrate the pre-existence of HBV NUCr substitution, which may largely attribute to the true naturally occurring substitutions and reflect the natural evolution characteristics of HBV variants with NUCr-related substitutions.

The acquisition of high-quality data is critical to a superior GenBank-based data mining study. After a rigorous screening process, 394 HBV RT sequences belonging to genotype A to H from 25 countries in 4 continents were recruited for this study, which showed a wide range of data sources. Moreover, a codon-based rather than a sequence-based method was used to calculate the substitution frequency because qualified HBV RT sequences had different lengths. This strategy could ensure not only an accurate calculation of the substitution frequency at each studied codon, but also an effective use of the available information.

In this study, AA substitutions at NUCr-related codons of RT were found in 72.7% (8/11) of the studied codons with frequencies ranging from 0.3% to 2.5% (Table 2). Secondary substitutions (1.6%) occurred more frequently than primary NUCr substitutions (0.5%). Interestingly, 59.3% (16/27) had an atypical AA substitution type at these codons, meaning that their roles in NUCr and viral fitness are still unknown, as is the case for rtM204L. This phenomenon was more obvious at the primary NUCr substitution codons (81.8%, 9/11) than at the secondary substitution codons (43.8%, 7/16) (Table 2). Therefore, our findings highly suggested that HBV could naturally mutate at these codons free of NUCs, but those variants carrying typical primary NUCr substitution(s) hardly evolve, probably due to their reduced survival fitness [2].

Our results show that only 11 sequences (2.8%) had typical NUCr-related substitutions. Substitutions rtL80V (*n* = 7) or rtV173L (*n* = 2) are typical secondary substitutions that can restore functional defects of HBV polymerase caused by primary LAM or LdT resistance substitutions [2,4,11]. These naturally occurring secondary substitutions might reduce the resistance barrier to LAM or LdT [2]. Substitution rtT184A/S (*n* = 2) was the only naturally occurring substitution detected as belonging to a typical primary NUCr substitution. It has been related to ETV resistance in the presence of the rtM204V + rtL180M backbone [73]. However, rtT184A/S alone would not lead to clinical resistance to ETV [74,75].

In addition, we found that the detected atypical substitutions of rtI169L, rtA181G, rtS202T/R, rtM204L, and rtM250K were not reported in NUCs-treated patients by the Stanford HBV Drug Resistance Database (https://hivdb.stanford.edu/HBV/HBVseq/development/HBVseq.html; accessing date: 17 January 2017). This suggests that these atypical substitutions might not play important roles in the emergence of NUCr as compared to rtA181T/V, rtS202C/G/I, rtM204I/V and rtM250I/L/V [2,76,77]. Consistent with our findings, atypical substitutions of rtI169L, rtS202R, and rtM204L have been detected in six treatment-naïve CHB patients with low proportions using a high-throughput sequencing method [78]. Kim et al. found that three patients had an rtI169L substitution at a level of 1–8% using a clone sequencing method [79]. We speculate that the reasons for the presence of such atypical substitutions might be due to a quasispecies property of HBV or sequencing errors [80,81,82]. Moreover, some variants might not be necessarily viable or infectious. Current evidence suggests that these atypical pre-existing variants have a limited role in the development of NUCr. Tenney et al. reported that substitution at rtI169 could only modestly decrease the susceptibility to ETV in the recombinant viruses with LAM resistance and rtM250V substitutions, increasing the viral fitness for survival [74]. Furthermore, in vitro studies showed that substitutions rtS202R and rtM250K would severely impair the replication capability of HBV variants with LAM resistance substitutions (rtL180M + rtM204V), while substitution rtS202T played only a minor role in ETV resistance [77]. Substitution rtM204L was shown to be sensitive to LAM in a study [83]. No study has been performed to elucidate the relationship between substitution rtA181G and ADV resistance.

Except for substitutions detected at eight codons, our data showed no substitutions at rtA194, rtN236 and rtL180. Since there were only 155 AAs at rtN236 available for analysis, this data might not be as solid as for rtA194 and rtL180, each of which had 332 and 355 available AAs, respectively (Figure 2). Moreover, by searching the Stanford HBV Drug Resistance Database, we found that rtA194, rtN236 and rtL180 in NUC treatment-naïve patients had very low substitution frequencies at 0.4% (14/3218), 0.03% (1/3218) and 0.2% (6/3218), respectively [84]. Considering that HBV RT sequences in this database were obtained both before and after 1998, our data together with their data suggested that the true naturally occurring substitutions at these codons should be very low.

We also found that genotype C sequences had the highest substitution frequency (16/122, 13.1%) at NUCr-related codons. However, we should notice that only 7 of 16 sequences harbored typical NUCr AA substitution types, i.e., 6 of rtL80V and 1 of rtT184A. Notably, 4 of 6 rtL80V-containing sequences were from different clones of the same patient. Thus, the naturally occurring NUCr substitutions in different HBV genotypes might not be as different as they seemed to be. This is in line with many previous drug resistance studies carried out after 1998 [2].

Although this study is novel in investigating the occurrence of NUCr substitutions before NUCs were approved, some limitations exist. Firstly, there was a limited number of sequences available in the GenBank database before 1998. Secondly, most sequences were from Asia, Europe and America (375/394, 95.2%) and data from Africa, a highly endemic area of HBV infection, is not adequate. Thirdly, some minor mutants in the quasispecies pool might not be detected due to the lack of a sensitive detection method. In fact, most substitutions (20/27, 74.1%) found in this study were determined by clone sequencing. Fourth, the possibility of sequencing errors or potential typos while submitting to GenBank could not be completely ruled out, especially for relatively rare AA substitutions, although the original studies submitting these sequences have been carefully reviewed.

In conclusion, we confirmed the presence of naturally occurring typical NUCr substitutions of rtL80V, rtV173L and rtT184A/S with low frequencies before the clinical approval of NUCs. Atypical substitutions of rtI169L, rtA181G, rtS202T/R, rtM204L, rtM250K were also detected. However, more clinical studies are still needed to explore the impacts of these baseline NUCr-related substitutions on antiviral responses.

## Figures and Tables

**Table 1:** Summary of potential AA substitutions associated with NUCr in treated CHB patients.

Drugs	Primary NUCr Substitutions	Secondary Substitutions
LAM	**rtA181T/V** [2,4,11], rtA181S [27], **rtM204I/V** [2,4,11], rtM204Q/S [28,29]	rtH55R [30], **rtL80I/V** [2,4,11], rtS117F [31], rtT128N [32], rtW153Q [32], **rtV173L** [2,4,11], **rtL180M** [2,4,11], rtL180C [33], rtA200V [34], rtV207I [35], rtL217P [36], rtL229F [37], rtQ267H [38], rtL269I [39]
ADV	rtS78T [40], **rtA181T/V** [2,4,11], rtA181S [41], rtE218G [42], rtI233V [43], **rtN236T** [2,4,11], rtN236V [44]	rtL80I/V [45], rtV84M [46], rtS85A [46], rtV214A [47], rtL217R [48], rtP237H [47]
ETV	rtS78T [18], **rtI169T** [2,4,11], **rtA181T/V** [2,4,11], **rtT184A/C/F/G/I/L/M/S** [2,4,11], rtA186T [17], **rtS202C/G/I** [2,4,11], **rtM204I/V** [2,4,11], **rtM250I/V/L** [2,4,11]	rtI163V [17], rtS219A [49], rtY245H [49], rtS246T [50], rtL269I [39]
LdT	**rtM204I/V** [2,4,11], **rtA181T/V** [2,4,11]	
TDF/TAF	rtS78T [18], rtP177G [51], rtA181T/V [2,4,11], **rtA194T** [2,4,11], rtF249A [51]	

Classic NUCr AA substitutions recommended by several clinical practice guidelines and authoritative reviews are indicated in bold [2,4,5,11,12,13]. Plain text represents non-classic AA substitutions that occurred sporadically. AA: amino acid; ADV: adefovir dipivoxil; CHB: chronic hepatitis B; ETV: entecavir; LAM: lamivudine; LdT: telbivudine; NUCr: nucleos(t)ide analogue resistance; rt: reverse transcriptase; TDF: tenofovir disoproxil fumarate; TAF: tenofovir alafenamide fumarate.

**Table 2:** The AA substitutions identified at the primary NUCr and secondary substitution codons in RT-containing sequences.

Substitution Category	NUCr-Related Codons	Substitution Types	No. of Substituted AAs	No. of Available AAs	Substitution Frequency (%) ^1^
Primary	rtI169	L	2	366	0.5
NUCr	rtA181	G	1	355	0.3
substitution	rtT184	A ^3^, S ^3^	1, 1	355	0.6
	rtA194	ND	0	332	0
	rtS202	T, R	2, 1	325	0.9
	rtM204	L	2	324	0.6
	rtN236	ND	0	155	0
	rtM250	K	1	138	0.7
Subtotal	8 codons	6 codons, 8 types	11	2350	0.5 ^2^
Secondary	rtL80	V ^3^	7	288	2.4
substitution	rtV173	G, A, L ^3^	5, 2, 2	366	2.5
	rtL180	ND	0	355	0
Subtotal	3 codons	2 codons, 4 types	16	1008	1.6 ^2^
Total	11 codons	8 codons, 12 types	27	3358	0.8

^1^ The substitution frequency at a studied codon was calculated as the percentage of the numbers of substituted AAs with respect to the available AAs at this codon; ^2^ The substitution frequency was significantly higher for the secondary substitution codons (16/1008, 1.6%) as compared to the primary NUCr substitution codons (11/2350, 0.5%) (χ^2^ = 11.08, *p* = 0.0009). Chi-square test was used; ^3^ AA substitution types belonged to the well-known typical NUCr substitutions. AA: amino acid; ND: not detected; NUCr: nucleos(t)ide analogue resistance; RT: reverse transcriptase.

**Table 3:** Characteristics of the RT sequences with NUCr-related AA substitutions.

Accession Number	Releasing Year	Sample Origin	Sequencing Method	Sequence Length (bp)	RT Coverage (AA)	Genotype	Substitution	References
X01587.1	1983	Japan	Clone	3214	1–344	C	V173G	[58]
M23808.1	1985	Japan	Clone	681	10–235	C	V173G	[59]
E01164.1	1986	Japan	Clone	748	6–253	C	V173G	NA
M38636.1	1988	South Korea	Clone	3213	1–344	C	V173G	[60]
U19777.1	1988	China	Clone	731	1–243	C	T184A	NA
M27765.1	1989	Japan	Clone	678	10–234	C	I169L	[61]
X14193.1	1989	South Korea	Clone	3213	1–344	C	V173G	[62]
S50225.1	1992	USA	Clone	3222	1–344	A	V173A	[63]
S56208.1	1992	India	Clone	123	169–208	D	S202T	[64]
X77310.1	1994	Italy	Direct	1401	1–300	D	S202R	NA
X80925.1	1995	UK	Direct	3182	1–344	D	S202T	[65]
D50519.1	1996	Japan	Clone	3215	1–344	C	A181G	[66]
AF013631.1	1997	China	Clone	681	10–235	C	L80V	[67]
AF036236.1	1997	China	Clone	838	1–236	C	L80V	NA
AF036237.1	1997	China	Clone	838	1–236	C	L80V	NA
AF036238.1	1997	China	Clone	838	1–236	C	L80V	NA
AF036239.1	1997	China	Clone	838	1–236	C	L80V	NA
U87739.1	1997	South Africa	Direct	846	1–235	A	L80V	[68]
AB014382.1	1998	Japan	Direct	3215	1–344	C	L80V	[69]
AJ003027.1	1998	Germany	Clone	1158	1–235	D	I169L	[70]
AF043564.1	1998	Argentina	Direct	217	122–193	A	V173A	[71]
AJ003026.1	1998	Germany	Clone	1164	1–235	D	V173L	[70]
AJ003028.1	1998	Germany	Clone	1164	1–235	D	V173L	[70]
AF044985.1	1998	Belgium	Clone	297	117–215	B	T184S	NA
AF074449.1	1998	Thailand	Direct	720	10–248	C	M204L	[72]
AF075604.1	1998	Thailand	Direct	720	10–248	C	M204L	NA
AJ012207.1	1998	Germany	Clone	3221	1–344	A	M250K	NA

AA: amino acid; bp: base pair; NA: not available; NUCr: nucleos(t)ide analogue resistance; RT: reverse transcriptase; UK: the United Kingdom of Great Britain and Northern Ireland; USA: the United States of America.

## Supplementary Materials

The following are available online at www.mdpi.com/1999-4915/9/8/199/s1, Figure S1: HBV genotypes identified in this study and their geographical distributions; Figure S2: The HBV sequences with recombinant genotypes found in this study; Figure S3: The distribution of HBV RT sequences with NUCr-related substitutions in different countries; Table S1: Summary of the relevant information of HBV sequences used in this study.
