# Supplementary material for: HBV Drug Resistance Substitutions Existed before the Clinical Approval of Nucleos(t)ide Analogues: A Bioinformatic Analysis by GenBank Data Mining
Source: Viruses. 2017 Jul 27;9(8):199. doi: 10.3390/v9080199 (PMC5580456; doi:10.3390/v9080199)
Supplement: Supplementary file 1 [file viruses-09-00199-s001.pdf]

Viruses **2016**, *8*, x; doi: www.mdpi.com/journal/viruses

Supplementary Materials: HBV Drug Resistance Substitutions Existed before the Clinical Approval of Nucleos(t)ide Analogues: A Bioinformatic Analysis by GenBank Data Mining

Xizhan Xu, Kuanhui Xiang, Mingze Su, Yao Li, Wei Ji, Yutang Li, Hui Zhuang and Tong Li

Table S1. Summary of the relevant information of hepatitis B virus (HBV) sequences used in this study.

GenBank Accession Number	Releasing Year	Country	Genotype	Nucleotide Sequence Length (bp)	RT Coverage (AA Sites)	Category [1]
J02202.1	1979	USA	D	3182	1–344	Full-length
NC_003977.2	1979	USA	D	3182	1–344	Full-length
J02205.1	1979	USA	A	892	1–267	Partial-length
X02763.1	1980	USA	A	3221	1–344	Full-length
E00010.1	1981	Japan	A	3221	1–344	Full-length
E00120.1	1981	Japan	A	3221	1–344	Full-length
A01865.1	1981	USA	D	681	10–235	Partial-length
E00070.1	1982	Japan	A	3221	1–344	Full-length
V00866.1	1983	Japan	A	3221	1–344	Full-length
D00630.1	1983	Japan	C	3215	1–344	Full-length
X01587.1	1983	Japan	C	3215	1–344	Full-length
E00121.1	1983	Japan	A	3221	1–344	Full-length
M54892.1	1984	China	C	3215	1–344	Full-length
M12393.1	1984	USA	D	829	1–241	Partial-length
X02496.1	1985	Germany	D	3182	1–344	Full-length
M23805.1	1985	Japan	C	681	10–235	Partial-length
M23806.1	1985	Japan	C	681	10–235	Partial-length
M23808.1	1985	Japan	C	681	10–235	Partial-length
M23809.1	1985	Japan	C	681	10–235	Partial-length
M23807.1	1985	Japan	C	681	10–235	Partial-length
V00867.1	1985	Japan	C	3215	1–344	Full-length
E00986.1	1986	Japan	C	858	1–237	Partial-length
X04615.1	1986	Japan	C	3215	1–344	Full-length
E01164.1	1986	USA	C	748	6–253	Partial-length
A32699.1	1986	USA	C	858	1–237	Partial-length
M38454.1	1987	China	C	3215	1–344	Full-length
M54898.1	1987	China (Taiwan)	A	3221	1–344	Full-length
M54923.1	1987	Indonesia	B	3215	1–344	Full-length
M17688.1	1987	Japan	C	678	10–234	Partial-length
M17549.1	1987	Japan	C	678	10–234	Partial-length
M17687.1	1987	Japan	C	678	10–234	Partial-length
M17550.1	1987	Japan	C	678	10–234	Partial-length

Table S1. *Cont.*

GenBank Accession Number	Releasing Year	Country	Genotype	Nucleotide Sequence Length (bp)	RT Coverage (AA Sites)	Category [1]
X04820.1	1987	Japan	A	681	10–235	Partial-length
M26141.1	1987	Japan	C	681	10–235	Partial-length
U19777.1	1988	China	C	731	1–243	Partial-length
E01498.1	1988	Japan	C	845	1–234	Partial-length
E01499.1	1988	Japan	C	817	2–234	Partial-length
D00329.1	1988	Japan	B	3215	1–344	Full-length
D00331.1	1988	Japan	B	3215	1–344	Full-length
D00330.1	1988	Japan	B	3215	1–344	Full-length
M21030.1	1988	Latvia	A	681	10–235	Partial-length
M57663.2	1988	Philippines	A	3221	1–344	Full-length
M38636.1	1988	South Korea	C	3215	1–344	Full-length
X14193.1	1988	South Korea	C	3215	1–344	Full-length
M27765.1	1989	Japan	C	678	10–234	Partial-length
M27766.1	1989	Japan	C	678	10–234	Partial-length
M30451.1	1989	USA	A	172	132–187	Partial-length
M32138.1	1990	France	D	3182	1–344	Full-length
X52939.1	1990	Germany	C	3215	1–344	Full-length
A29025.1	1990	Japan	A	860	1–237	Partial-length
A29023.1	1990	Japan	C	848	1–237	Partial-length
U19779.1	1991	China	C	72	10–32	Partial-length
M74500.1	1991	France	A/G [2]	1203	1–235	Partial-length
M74498.1	1991	France	A	1203	1–235	Partial-length
M74499.1	1991	France	G	1203	1–235	Partial-length
X59795.1	1991	Italy	D	3182	1–344	Full-length
E02707.1	1991	Japan	C	561	1–185	Partial-length
D10055.1	1991	Japan	C	846	1–235	Partial-length
S56208.1	1992	India	D	123	169–208	Partial-length
X65259.1	1992	Italy	D	3182	1–344	Full-length
X68292.1	1992	Italy	D/A [2]	3182	1–344	Full-length
X65258.1	1992	Italy	D/A [2]	3182	1–344	Full-length
X65257.1	1992	Italy	D	3182	1–344	Full-length
D12980.1	1992	Japan	C	3215	1–344	Full-length
A18244.1	1992	USA	A	585	41–234	Partial-length
A18226.1	1992	USA	D	822	1–234	Partial-length
A18243.1	1992	USA	D	678	10–234	Partial-length
S50225.1	1992	USA	A	3221	1–344	Full-length
X69458.1	1992	Zimbabwe	A	1232	1–239	Partial-length
L24071.1	1993	Gambia	E	681	10–235	Partial-length
X70185.1	1993	Germany	A	3221	1–344	Full-length

Table S1. *Cont.*

GenBank Accession Number	Releasing Year	Country	Genotype	Nucleotide Sequence Length (bp)	RT Coverage (AA Sites)	Category [1]
X69798.1	1993	Germany	F	3215	1–344	Full-length
A08967.1	1993	Germany	D	3182	1–344	Full-length
X72702.1	1993	Germany	D	3182	1–344	Full-length
S62754.1	1993	Japan	C	1203	1–235	Partial-length
D16665.1	1993	Japan	C	3215	1–344	Full-length
X75660.1	1993	Sweden	B	681	10–235	Partial-length
X75669.1	1993	Sweden	A	681	10–235	Partial-length
X75666.1	1993	Sweden	A	681	10–235	Partial-length
X75662.1	1993	Sweden	D	681	10–235	Partial-length
X75792.1	1993	Sweden	C	681	10–235	Partial-length
X75667.1	1993	Sweden	C	681	10–235	Partial-length
X75668.1	1993	Sweden	D	681	10–235	Partial-length
X75661.1	1993	Sweden	F	681	10–235	Partial-length
L08805.1	1993	USA	C	3215	1–344	Full-length
S74815.1	1994	China	B	3215	1–344	Full-length
S74867.1	1994	China	B	3215	1–344	Full-length
X77308.1	1994	Italy	D/A [2]	1171	1–228	Partial-length
X77310.1	1994	Italy	D	1401	1–300	Partial-length
X77309.1	1994	Italy	D	1171	1–223	Partial-length
D23680.1	1994	Japan	B	3215	1–344	Full-length
D23681.1	1994	Japan	B	3215	1–344	Full-length
D23682.1	1994	Japan	B	3215	1–344	Full-length
D23683.1	1994	Japan	B	3215	1–344	Full-length
D23684.1	1994	Japan	B	3215	1–344	Full-length
S75184.1	1994	Japan	C	3215	1–344	Full-length
D23679.1	1994	Japan	C	3215	1–344	Full-length
D23677.1	1994	Japan	C	3215	1–344	Full-length
D28880.1	1994	Japan	C	3215	1–344	Full-length
D23678.1	1994	Japan	C	3215	1–344	Full-length
Z35717.1	1994	Poland	A	3221	1–344	Full-length
Z35716.1	1994	Poland	D	3182	1–344	Full-length
X75657.1	1994	Sweden	E	3212	1–344	Full-length
X75664.1	1994	Sweden	E	3212	1–344	Full-length
X75658.1	1994	Sweden	F	3215	1–344	Full-length
X75663.1	1994	Sweden	F	3215	1–344	Full-length
X75656.1	1994	Sweden	C	3215	1–344	Full-length
X75665.1	1994	Sweden	C	3215	1–344	Full-length
L29017.1	1994	UK	E	681	10–235	Partial-length
L27106.1	1994	USA	D	3182	1–344	Full-length

Table S1. *Cont.*

GenBank Accession Number	Releasing Year	Country	Genotype	Nucleotide Sequence Length (bp)	RT Coverage (AA Sites)	Category [1]
AF013630.1	1995	China	B	681	10–235	Partial-length
AF013629.1	1995	China	B	681	10–235	Partial-length
X85254.1	1995	Italy	D	3182	1–344	Full-length
D50489.1	1995	Japan	C	3215	1–344	Full-length
D16667.1	1995	Japan	C	3215	1–344	Full-length
D16666.1	1995	Japan	C	3215	1–344	Full-length
X80924.1	1995	UK	D	3182	1–344	Full-length
X80925.1	1995	UK	D	3182	1–344	Full-length
X80926.1	1995	UK	D	3182	1–344	Full-length
U50174.1	1996	Argentina	A	210	119–187	Partial-length
U50176.1	1996	Argentina	A	210	119–187	Partial-length
U50177.1	1996	Argentina	A	210	119–187	Partial-length
U50175.1	1996	Argentina	B	210	119–187	Partial-length
U50171.1	1996	Argentina	D	210	119–187	Partial-length
U50173.1	1996	Argentina	A	210	119–187	Partial-length
U50170.1	1996	Argentina	D	210	119–187	Partial-length
U50180.1	1996	Argentina	F	210	119–187	Partial-length
U50169.1	1996	Argentina	F	210	119–187	Partial-length
U50179.1	1996	Argentina	F	210	119–187	Partial-length
U50172.1	1996	Argentina	F	210	119–187	Partial-length
U50178.1	1996	Argentina	F	210	119–187	Partial-length
U55223.1	1996	Brazil	D	1149	1–228	Partial-length
U55226.1	1996	Brazil	D	1149	1–228	Partial-length
U55225.1	1996	Brazil	D	1149	1–228	Partial-length
U55224.1	1996	Brazil	D	1149	1–228	Partial-length
U55227.1	1996	Brazil	D	1149	1–228	Partial-length
U55228.1	1996	Brazil	D	1149	1–228	Partial-length
U55220.1	1996	Brazil	A	1149	1–228	Partial-length
U55222.1	1996	Brazil	A	1149	1–228	Partial-length
U55221.1	1996	Brazil	A	1149	1–228	Partial-length
Z72478.1	1996	Germany	A	3221	1–344	Full-length
Z72479.1	1996	Germany	A	3221	1–344	Full-length
X51970.1	1996	Germany	A	3221	1–344	Full-length
S81946.1	1996	Japan	C	1155	1–235	Partial-length
S81945.1	1996	Japan	C	1203	1–235	Partial-length
E10905.1	1996	Japan	A	3221	1–344	Full-length
D50521.1	1996	Japan	B	3215	1–344	Full-length
D50522.1	1996	Japan	B	3215	1–344	Full-length
D50517.1	1996	Japan	C	3215	1–344	Full-length

Table S1. *Cont.*

GenBank Accession Number	Releasing Year	Country	Genotype	Nucleotide Sequence Length (bp)	RT Coverage (AA Sites)	Category [1]
D50519.1	1996	Japan	C	3215	1–344	Full-length
X98076.1	1996	Switzerland	B	3215	1–344	Full-length
X97848.1	1996	UK	D	3182	1–344	Full-length
X97849.1	1996	UK	D	3182	1–344	Full-length
X97850.1	1996	UK	B	3215	1–344	Full-length
X97851.1	1996	UK	B	3215	1–344	Full-length
U91807.1	1997	Central America	F	681	10–235	Partial-length
U91826.1	1997	Central America	F	681	10–235	Partial-length
U91824.1	1997	Central America	A	681	10–235	Partial-length
U91832.1	1997	Central America	D	681	10–235	Partial-length
U91804.1	1997	Central America	D	681	10–235	Partial-length
U91812.1	1997	Central America	F	681	10–235	Partial-length
U91806.1	1997	Central America	F	681	10–235	Partial-length
U91830.1	1997	Central America	F	681	10–235	Partial-length
U91816.1	1997	Central America	D	678	10–234	Partial-length
U91823.1	1997	Central America	F	681	10–235	Partial-length
U91820.1	1997	Central America	A	681	10–235	Partial-length
U91810.1	1997	Central America	A	681	10–235	Partial-length
U91827.1	1997	Central America	H	681	10–235	Partial-length
U91818.1	1997	Central America	F	681	10–235	Partial-length
U91805.1	1997	Central America	F	681	10–235	Partial-length
U91815.1	1997	Central America	F	681	10–235	Partial-length
U91813.1	1997	Central America	F	681	10–235	Partial-length
U91809.1	1997	Central America	D	681	10–235	Partial-length
U91831.1	1997	Central America	F	681	10–235	Partial-length
U91829.1	1997	Central America	F	681	10–235	Partial-length
U91828.1	1997	Central America	F	681	10–235	Partial-length
U91821.1	1997	Central America	F	681	10–235	Partial-length
U91825.1	1997	Central America	F	681	10–235	Partial-length
U91811.1	1997	Central America	F	681	10–235	Partial-length
U91803.1	1997	Central America	F	681	10–235	Partial-length
U91814.1	1997	Central America	F	681	10–235	Partial-length
U91808.1	1997	Central America	F	681	10–235	Partial-length
U91819.1	1997	Central America	H	681	10–235	Partial-length
U91822.1	1997	Central America	F	681	10–235	Partial-length
U91817.1	1997	Central America	F	681	10–235	Partial-length
AF036238.1	1997	China	C	838	1–236	Partial-length
AF036236.1	1997	China	C	838	1–236	Partial-length
AF036237.1	1997	China	C	838	1–236	Partial-length

Table S1. *Cont.*

GenBank Accession Number	Releasing Year	Country	Genotype	Nucleotide Sequence Length (bp)	RT Coverage (AA Sites)	Category [1]
AF036239.1	1997	China	C	838	1–236	Partial-length
AF013631.1	1997	China	C	681	10–235	Partial-length
AF065111.1	1997	Germany	A	681	10–235	Partial-length
Y07587.1	1997	Germany	D	3182	1–344	Full-length
U87727.1	1997	South Africa	A	846	1–235	Partial-length
U87736.1	1997	South Africa	A	846	1–235	Partial-length
U87729.1	1997	South Africa	A	846	1–235	Partial-length
U87728.1	1997	South Africa	A	846	1–235	Partial-length
U87733.1	1997	South Africa	A	846	1–235	Partial-length
U87734.1	1997	South Africa	A	846	1–235	Partial-length
U87726.1	1997	South Africa	A	846	1–235	Partial-length
U87732.1	1997	South Africa	A	846	1–235	Partial-length
U87731.1	1997	South Africa	A	846	1–235	Partial-length
U87739.1	1997	South Africa	A	846	1–235	Partial-length
U87735.1	1997	South Africa	A	846	1–235	Partial-length
U87730.1	1997	South Africa	A	846	1–235	Partial-length
U87737.1	1997	South Africa	D	846	1–235	Partial-length
U87725.1	1997	South Africa	A	846	1–235	Partial-length
U87851.1	1997	South Africa	D/A [2]	846	1–235	Partial-length
U87738.1	1997	South Africa	D	846	1–235	Partial-length
U88095.1	1997	South Africa	A	681	10–235	Partial-length
X98072.1	1997	Switzerland	B	3215	1–344	Full-length
X98073.1	1997	Switzerland	B	3215	1–344	Full-length
X98074.1	1997	Switzerland	B	3215	1–344	Full-length
X98075.1	1997	Switzerland	B	3215	1–344	Full-length
X98077.1	1997	Switzerland	B	3215	1–344	Full-length
U95551.1	1997	USA	D	3182	1–344	Full-length
AF043575.1	1998	Argentina	A	210	119–187	Partial-length
AF043567.1	1998	Argentina	A	222	121–194	Partial-length
AF043569.1	1998	Argentina	F	215	124–194	Partial-length
AF043559.1	1998	Argentina	A	214	122–191	Partial-length
AF043576.1	1998	Argentina	A	213	122–191	Partial-length
AF043562.1	1998	Argentina	A	222	122–194	Partial-length
AF043566.1	1998	Argentina	A	217	123–194	Partial-length
AF043579.1	1998	Argentina	A	256	123–207	Partial-length
AF043571.1	1998	Argentina	A	213	123–192	Partial-length
AF043568.1	1998	Argentina	A	216	123–194	Partial-length
AF043572.1	1998	Argentina	D	217	123–194	Partial-length
AF043570.1	1998	Argentina	A	248	122–203	Partial-length

Table S1. *Cont.*

GenBank Accession Number	Releasing Year	Country	Genotype	Nucleotide Sequence Length (bp)	RT Coverage (AA Sites)	Category [1]
AF043563.1	1998	Argentina	A	222	122–193	Partial-length
AF043565.1	1998	Argentina	F	222	122–194	Partial-length
AF043564.1	1998	Argentina	A	222	122–193	Partial-length
AF043561.2	1998	Argentina	F	675	10–233	Partial-length
AF043573.2	1998	Argentina	F	678	10–234	Partial-length
AF061523.1	1998	Argentina	D	681	10–235	Partial-length
AF043578.2	1998	Argentina	F	678	10–234	Partial-length
AF043577.2	1998	Argentina	F	678	10–234	Partial-length
AF043560.2	1998	Argentina	A	3221	1–344	Full-length
AF043580.2	1998	Argentina	A	3221	1–344	Full-length
AF044989.1	1998	Belgium	B	297	117–215	Partial-length
AF044992.1	1998	Belgium	C	297	117–215	Partial-length
AF045004.1	1998	Belgium	E	297	117–215	Partial-length
AF044996.1	1998	Belgium	C	297	117–215	Partial-length
AF044987.1	1998	Belgium	B	297	117–215	Partial-length
AF044991.1	1998	Belgium	C	297	117–215	Partial-length
AF044986.1	1998	Belgium	B	297	117–215	Partial-length
AF044985.1	1998	Belgium	B	297	117–215	Partial-length
AF045007.1	1998	Belgium	E	297	117–215	Partial-length
AF044982.1	1998	Belgium	A	297	117–215	Partial-length
AF044993.1	1998	Belgium	C	297	117–215	Partial-length
AF045003.1	1998	Belgium	E	297	117–215	Partial-length
AF044988.1	1998	Belgium	B	297	117–215	Partial-length
AF044990.1	1998	Belgium	C	297	117–215	Partial-length
AF044994.1	1998	Belgium	C	297	117–215	Partial-length
AF045005.1	1998	Belgium	E	297	117–215	Partial-length
AF045006.1	1998	Belgium	E	297	117–215	Partial-length
AF044995.1	1998	Belgium	C	297	117–215	Partial-length
AF045010.1	1998	Belgium	F	297	117–215	Partial-length
AF044979.1	1998	Belgium	A	297	117–215	Partial-length
AF045012.1	1998	Belgium	F	297	117–215	Partial-length
AF045000.1	1998	Belgium	D	297	117–215	Partial-length
AF045002.1	1998	Belgium	D	297	117–215	Partial-length
AF045011.1	1998	Belgium	F	297	117–215	Partial-length
AF044983.1	1998	Belgium	A	297	117–215	Partial-length
AF044980.1	1998	Belgium	A	297	117–215	Partial-length
AF045013.1	1998	Belgium	F	297	117–215	Partial-length
AF044997.1	1998	Belgium	D	297	117–215	Partial-length
AF044984.1	1998	Belgium	A	297	117–215	Partial-length

Table S1. *Cont.*

GenBank Accession Number	Releasing Year	Country	Genotype	Nucleotide Sequence Length (bp)	RT Coverage (AA Sites)	Category [1]
AF045001.1	1998	Belgium	D	297	117–215	Partial-length
AF044998.1	1998	Belgium	D	297	117–215	Partial-length
AF044999.1	1998	Belgium	D	297	117–215	Partial-length
AF045008.1	1998	Belgium	F	297	117–215	Partial-length
AF044981.1	1998	Belgium	A	297	117–215	Partial-length
AF045009.1	1998	Belgium	F	297	117–215	Partial-length
AJ005283.1	1998	China	C	558	1–185	Partial-length
AF052576.1	1998	China	C	1203	1–235	Partial-length
AF042065.1	1998	China (Taiwan)	C	230	112–187	Partial-length
AF043574.1	1998	China (Taiwan)	A	206	124–191	Partial-length
AF061525.1	1998	Germany	A	681	10–235	Partial-length
AF061526.1	1998	Germany	A	681	10–235	Partial-length
AF065116.1	1998	Germany	A	681	10–235	Partial-length
AF065112.1	1998	Germany	D	681	10–235	Partial-length
AF065113.1	1998	Germany	A	681	10–235	Partial-length
AF065110.1	1998	Germany	D	681	10–235	Partial-length
AF065118.1	1998	Germany	D	681	10–235	Partial-length
AF065114.1	1998	Germany	A	681	10–235	Partial-length
AF065117.1	1998	Germany	D	681	10–235	Partial-length
AF065119.1	1998	Germany	D	681	10–235	Partial-length
AF061527.1	1998	Germany	B	681	10–235	Partial-length
AF061528.1	1998	Germany	D	681	10–235	Partial-length
AF065115.1	1998	Germany	A	681	10–235	Partial-length
AF061524.1	1998	Germany	D	681	10–235	Partial-length
AJ003027.1	1998	Germany	D	1158	1–235	Partial-length
AJ003116.1	1998	Germany	D	1170	1–235	Partial-length
AJ003028.1	1998	Germany	D	1164	1–235	Partial-length
AJ003026.1	1998	Germany	D	1164	1–235	Partial-length
AJ012207.1	1998	Germany	A	3221	1–344	Full-length
AF043593.1	1998	Germany	D	3182	1–344	Full-length
AF043594.1	1998	Germany	D	3182	1–344	Full-length
AF041251.1	1998	Iran	D	681	10–235	Partial-length
AB014381.1	1998	Japan	C	3215	1–344	Full-length
AB014385.1	1998	Japan	C	3215	1–344	Full-length
AB014366.1	1998	Japan	C	3215	1–344	Full-length
AB014371.1	1998	Japan	C	3215	1–344	Full-length
AB014393.1	1998	Japan	C	3215	1–344	Full-length
AB014396.1	1998	Japan	C	3215	1–344	Full-length
AB014370.1	1998	Japan	C	3215	1–344	Full-length

Table S1. *Cont.*

GenBank Accession Number	Releasing Year	Country	Genotype	Nucleotide Sequence Length (bp)	RT Coverage (AA Sites)	Category [1]
AB014372.1	1998	Japan	C	3215	1–344	Full-length
AB014399.1	1998	Japan	C	3215	1–344	Full-length
AB014360.1	1998	Japan	C	3215	1–344	Full-length
AB014382.1	1998	Japan	C	3215	1–344	Full-length
AB014383.1	1998	Japan	C	3215	1–344	Full-length
AB014384.1	1998	Japan	C	3215	1–344	Full-length
AB014364.1	1998	Japan	C	3215	1–344	Full-length
AB014386.1	1998	Japan	C	3215	1–344	Full-length
AB014380.1	1998	Japan	C	3215	1–344	Full-length
AB014392.1	1998	Japan	C	3215	1–344	Full-length
AB014389.1	1998	Japan	C	3215	1–344	Full-length
AB014369.1	1998	Japan	C	3215	1–344	Full-length
AB014391.1	1998	Japan	C	3215	1–344	Full-length
AB014394.1	1998	Japan	C	3215	1–344	Full-length
D50520.1	1998	Japan	C	3215	1–344	Full-length
AB014374.1	1998	Japan	C	3215	1–344	Full-length
D50518.1	1998	Japan	C	3215	1–344	Full-length
AB014362.1	1998	Japan	C	3215	1–344	Full-length
AB014363.1	1998	Japan	C	3215	1–344	Full-length
AB014378.1	1998	Japan	C	3215	1–344	Full-length
AB014365.1	1998	Japan	C	3215	1–344	Full-length
AB014367.1	1998	Japan	C	3215	1–344	Full-length
AB014379.1	1998	Japan	C	3215	1–344	Full-length
AB014376.1	1998	Japan	C	3215	1–344	Full-length
AB014377.1	1998	Japan	C	3215	1–344	Full-length
AB014397.1	1998	Japan	C	3215	1–344	Full-length
AB014390.1	1998	Japan	C	3215	1–344	Full-length
AB014373.1	1998	Japan	C	3215	1–344	Full-length
AB014387.1	1998	Japan	C	3215	1–344	Full-length
AB014398.1	1998	Japan	C	3215	1–344	Full-length
AB014361.1	1998	Japan	C	3215	1–344	Full-length
AB014375.1	1998	Japan	C	3215	1–344	Full-length
AB014388.1	1998	Japan	C	3215	1–344	Full-length
AB014368.1	1998	Japan	C	3215	1–344	Full-length
AB014395.1	1998	Japan	C	3215	1–344	Full-length
AF050293.1	1998	Philippines	B	159	113–165	Partial-length
AF050314.1	1998	Philippines	A	159	113–165	Partial-length
AF050295.1	1998	Philippines	A	159	113–165	Partial-length
AF050299.1	1998	Philippines	A	159	113–165	Partial-length

Table S1. *Cont.*

GenBank Accession Number	Releasing Year	Country	Genotype	Nucleotide Sequence Length (bp)	RT Coverage (AA Sites)	Category [1]
AF050317.1	1998	Philippines	A	159	113–165	Partial-length
AF050291.1	1998	Philippines	A	159	113–165	Partial-length
AF050313.1	1998	Philippines	A	159	113–165	Partial-length
AF050306.1	1998	Philippines	G	159	113–165	Partial-length
AF050304.1	1998	Philippines	A	159	113–165	Partial-length
AF050310.1	1998	Philippines	B	159	113–165	Partial-length
AF050298.1	1998	Philippines	A	159	113–165	Partial-length
AF050301.1	1998	Philippines	A	159	113–165	Partial-length
AF050308.1	1998	Philippines	A	159	113–165	Partial-length
AF050307.1	1998	Philippines	C	159	113–165	Partial-length
AF050316.1	1998	Philippines	A	159	113–165	Partial-length
AF050309.1	1998	Philippines	B	159	113–165	Partial-length
AF050297.1	1998	Philippines	A	159	113–165	Partial-length
AF050305.1	1998	Philippines	C	159	113–165	Partial-length
AF050303.1	1998	Philippines	A	159	113–165	Partial-length
AF050315.1	1998	Philippines	A	159	113–165	Partial-length
AF050302.1	1998	Philippines	A	159	113–165	Partial-length
AF050294.1	1998	Philippines	A	159	113–165	Partial-length
AF050296.1	1998	Philippines	A	159	113–165	Partial-length
AF050292.1	1998	Philippines	C	159	113–165	Partial-length
AF050312.1	1998	Philippines	A	159	113–165	Partial-length
AF050311.1	1998	Philippines	A	159	113–165	Partial-length
AF050300.1	1998	Philippines	A	159	113–165	Partial-length
AF074446.1	1998	Thailand	A	183	119–178	Partial-length
AF074447.1	1998	Thailand	C	183	119–178	Partial-length
AF074438.1	1998	Thailand	C	183	119–178	Partial-length
AF074448.1	1998	Thailand	C	183	119–178	Partial-length
AF074441.1	1998	Thailand	B	183	119–178	Partial-length
AF074440.1	1998	Thailand	C	183	119–178	Partial-length
AF074445.1	1998	Thailand	B	183	119–178	Partial-length
AF074439.1	1998	Thailand	B	183	119–178	Partial-length
AF074443.1	1998	Thailand	C	183	119–178	Partial-length
AF074442.1	1998	Thailand	A	183	119–178	Partial-length
AF074444.1	1998	Thailand	C	183	119–178	Partial-length
AF072465.1	1998	Thailand	C	720	10–248	Partial-length
AF072464.1	1998	Thailand	C	720	10–248	Partial-length
AF075604.1	1998	Thailand	C/A [2]	720	10–248	Partial-length
AF072466.1	1998	Thailand	C	720	10–248	Partial-length
AF074449.1	1998	Thailand	C	720	10–248	Partial-length

Table S1. *Cont.*

GenBank Accession Number	Releasing Year	Country	Genotype	Nucleotide Sequence Length (bp)	RT Coverage (AA Sites)	Category [1]
AF083634.1	1998	UK	A	681	10–235	Partial-length
AF083637.1	1998	UK	A	681	10–235	Partial-length
AF083641.1	1998	UK	D	681	10–235	Partial-length
AF083636.1	1998	UK	A	681	10–235	Partial-length
AF083640.1	1998	UK	D	681	10–235	Partial-length
AF083638.1	1998	UK	A	681	10–235	Partial-length
AF083635.1	1998	UK	A	681	10–235	Partial-length
AF083639.1	1998	UK	A	681	10–235	Partial-length
AF049109.1	1998	USA	A	416	100–238	Partial-length
L13994.1	1998	USA	A	3221	1–344	Full-length
AJ012481.1	1998	Vietnam	C	916	8–312	Partial-length

[1] "Partial-length" and "full-length" indicate partial-length and full-length RT sequence, respectively. [2] These six sequences were identified as recombinant genotypes. AA: amino acid; bp: base pair; RT: reverse transcriptase; UK: the United Kingdom of Great Britain and Northern Ireland; USA: the United States of America.

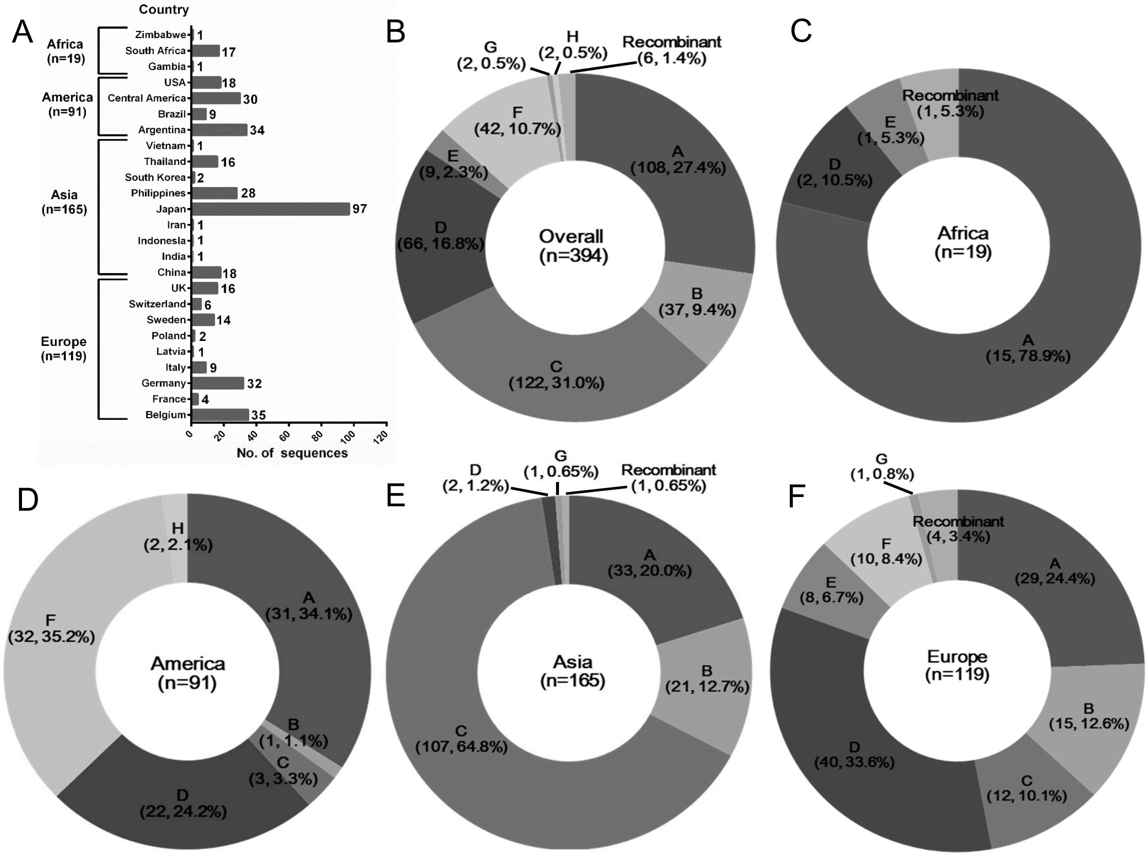

Figure S1. HBV genotypes identified in this study and their geographical distribution. (**A**) The distribution of 394 recruited HBV sequences in different countries. (**B–F**) Genotype distribution of the recruited sequences worldwide (**B**), in Africa (**C**), in America (**D**), in Asia (**E**) and in Europe (**F**). UK, the United Kingdom of Great Britain and Northern Ireland; USA, the United States of America.

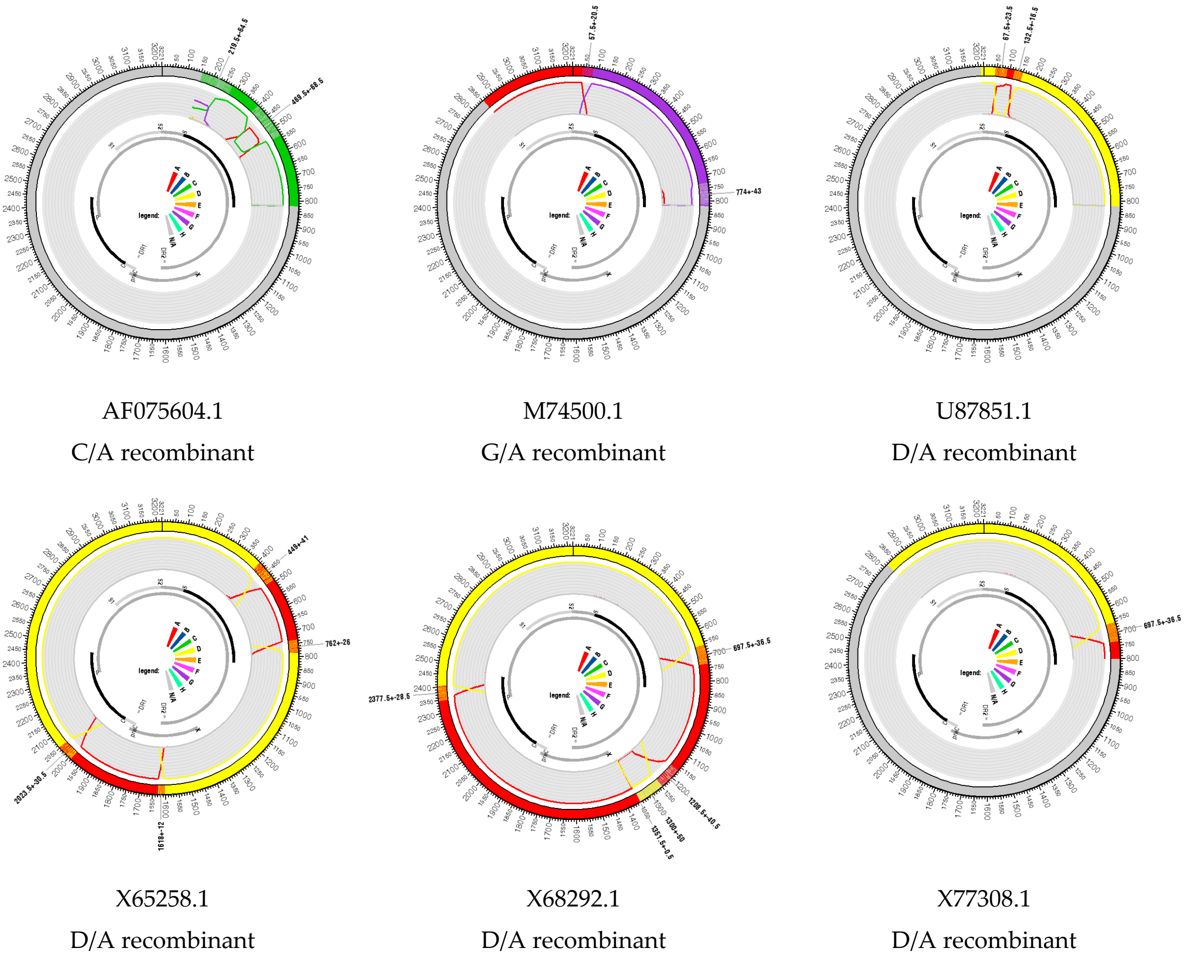

Figure S2. The HBV sequences with recombinant genotypes analyzed in this study. jpHMM tool was used for recombination analysis of HBV sequences. Sequences are color-coded by genotypes. Regions with a shading of two colors are marked with breakpoint interval.

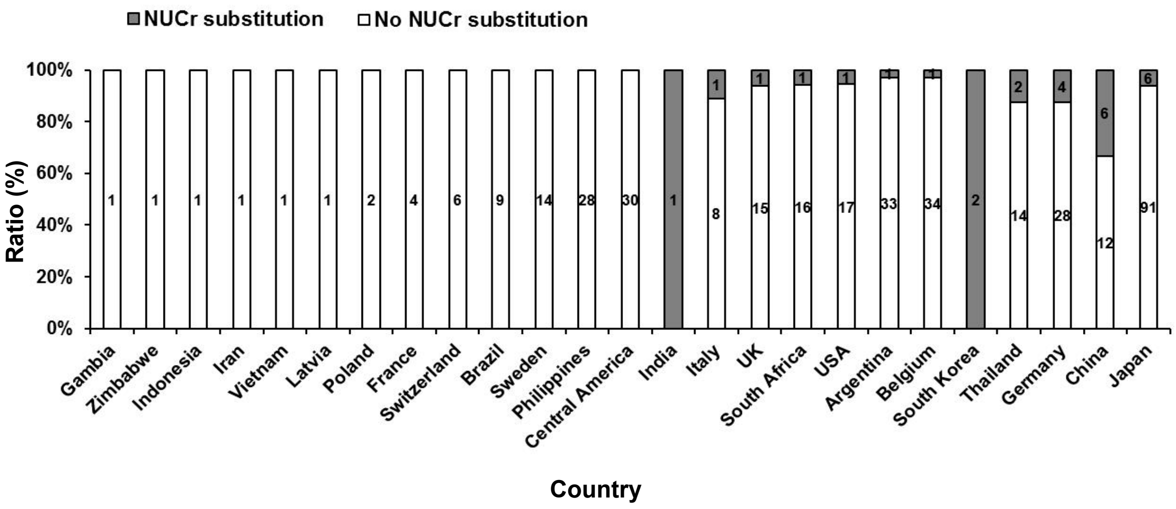

Figure S3. The distribution of HBV RT sequences with NUCr-related substitutions in different countries.

© 2017 by the authors; licensee MDPI, Basel, Switzerland. This article is an open access article distributed under the terms and conditions of the Creative Commons by Attribution (CC-BY) license (http://creativecommons.org/licenses/by/4.0/).
